# Apex Predators Enhance Environmental Adaptation but Reduce Community Stability of Bacterioplankton in Crustacean Aquaculture Ponds

**DOI:** 10.3390/ijms231810785

**Published:** 2022-09-15

**Authors:** Yiran Hou, Rui Jia, Bing Li, Jian Zhu

**Affiliations:** 1 Key Laboratory of Integrated Rice-Fish Farming Ecology, Ministry of Agriculture and Rural Affairs, Freshwater Fisheries Research Center, Chinese Academy of Fishery Sciences, Wuxi 214081, China; 2 Wuxi Fisheries College, Nanjing Agricultural University, Wuxi 214081, China

**Keywords:** apex predator, bacterioplankton, community assembly, community stability, crustacean aquaculture, environmental adaptation

## Abstract

Aquaculture environments harbor complex bacterial communities that are critical for the growth and health of culture species. Apex predators are frequently added to aquaculture ponds to improve ecosystem stability. However, limited research has explored the effects of apex predators on the composition and function of bacterioplankton communities, as well as the underlying mechanisms of community assembly. Using 16S ribosomal RNA (rRNA) high-throughput sequencing, we investigated bacterioplankton communities of crustacean aquaculture ponds with and without apex predators (mandarin fish, *Siniperca chuatsi*) throughout the culture process. In addition to investigating differences in bacterioplankton communities, we also explored variations in environmental adaptation, functional redundancy, and community stability. Significant differences were observed in bacterioplankton composition among different cultural stages; there was an increase in *Bacteriobota* and fermentation-related bacteria, but a decrease in Firmicutes and pathogens in the middle stages of aquaculture. Apex predators increased the abundance of organic matter degradation bacteria and decreased pathogens. Bacterioplankton communities under apex predator disturbances had a wider environmental breadth, indicating broader environmental adaptation. Moreover, functional prediction and network analyses revealed that communities under apex predator disturbances were less functionally redundant and unstable. Based on the null model, stochastic processes drove community assembly during aquaculture, whereas apex predators elevated the contribution of deterministic processes. Greater changes in nitrate in culture ponds caused by apex predator disturbances were decisive in controlling the balance between stochasticity and determinism in community assembly. Our study provided insight into the mechanisms underlying bacterioplankton community assembly in aquaculture systems in response to apex predator disturbances.

## 1. Introduction

The rising global human population increases the demand for food, promoting the rapid expansion of food industries. Aquatic products are fundamental food sources that have expanded in recent years [1]. More than 70% of aquatic products worldwide are produced in China, and freshwater aquaculture ponds are the main mode of production [2]. Hence, the aquaculture pond, as a typically aquatic ecosystem with artificial nutrient inputs, has become more and more important [2]. Some apex predator species have been added to aquaculture ponds to maintain a better culture environment and improve economic efficiency [3]. Apex predators occupy positions at the top of the food web and play key roles in ecosystem stability [4]. When added to crustacean aquaculture systems, they can prey upon old and weak animals, resulting in stronger, healthier animals that are less prone to disease outbreaks [5]. Microbial communities in aquaculture environments efficiently regulate nutrient cycling to maintain environmental quality [6]. However, the effects of apex predators on microbe-driven ecological processes and functions in aquaculture ecosystems are not fully understood.

Apex predators can alter environmental conditions via food webs, resulting in heterogeneity in aquaculture systems [7]. Environmental adaptation in a community reflects suitable conditions or environments for species’ lineage colonization and reproduction [8]. Specifically, the environmental breadth of a species is the range of environmental conditions in which it can survive [9]. By influencing species growth and propagation, environmental factors shape the microbial community by determining environmental breadth [10]. In addition, different microbes often have overlapping metabolic activities, resulting in high functional redundancy in the microbial community [11]. Specific environmental stresses can reduce the complexity of the microbial community and thereby impact its functional redundancy [12]. Furthermore, the microbial community tends to exhibit relative stability to resist environmental disturbances [13]. Community stability can be characterized by some features of molecular ecological networks (MENs), in which nodes represent species and links represent interactions between them [14]. In general, a high percentage of negative correlations in MENs indicates a more stable microbial community [15]. Previous studies have mainly focused on how environmental variation affects microbial community composition. However, we have less understanding of the effects of apex predator disturbances on the adaptation and stability of microbial communities.

Understanding the underlying mechanisms shaping community assembly is a key aim of microbial ecology research [16]. Bacterial community assembly is simultaneously controlled by deterministic and stochastic processes [17]. In extreme cases, the existence of each species in a habitat can be predicted, representing the community structure governed by environmental selection [18]. This community assembly process induced by abiotic and biotic factors is deterministic [19]. Alternatively, multiple species may exist in similar or overlapping habitats in natural environments, and specific species may not be eliminated through competition between species [20]. In this case, changes in the relative abundance of species in the community are caused by stochastic fluctuations [21]. Importantly, environmental factors play vital roles in regulating the homeostasis of deterministic and stochastic processes to impact bacterial community assembly [22]. For example, the available potassium is a key driver in rice fields, and it mediates the relative importance of deterministic and stochastic processes in soil bacterial community assembly [23]. Similarly, dissolved oxygen is the main factor influencing deterministic and stochastic governance in lake bacterioplankton community assembly [24]. Although some illuminating results have been reported, the assembly dynamics of bacterial communities and their relationships with environmental factors in aquaculture systems with apex predator disturbances remain unclear.

The objectives of the present study were: (i) to investigate the assembly dynamics of bacterioplankton communities in aquaculture systems affected by apex predator species, and (ii) to decipher the mechanism underlying the variation in bacterioplankton communities induced by apex predator disturbance. To achieve these goals, we performed high-throughput sequencing of 16S ribosomal RNA (rRNA) genes in the water of crustacean aquaculture ponds with and without apex predators throughout the culture process. Changes in the bacterioplankton community composition, function, and stability caused by apex predators were assessed. Regarding environmental factors, the effects of apex predator disturbances on the environmental adaptation of bacterioplankton communities were evaluated. The ecological processes shaping the assembly of bacterioplankton communities were also explored, and variation associated with apex predator disturbances was revealed. The findings expanded our understanding of bacterioplankton community assembly in crustacean aquaculture and could improve management strategies for the addition of apex predator species.

## 2. Results

### 2.1. Overview of Bacterioplankton Communities

We examined the bacterioplankton communities of MC and PC aquaculture ponds throughout the whole culture process. In total, 32 water samples were sequenced, generating 952,743 high-quality tags, ranging from 16,984 to 52,874 tags per sample. These tags were clustered into 4682 OTUs at the 97% similarity level. Four alpha diversity indices were calculated to assess bacterioplankton communities, and the results are shown in Figure S1. No significant differences were observed for any alpha diversity indices among bacterioplankton communities at different culture stages or between MC and PC ponds (Tukey’s HSD test, *p* > 0.05, Table S1). These results indicated that the diversity of bacterioplankton communities tended to remain stable during culturing, and apex predator disturbances had no apparent impact on it.

### 2.2. Variations in Bacterioplankton Communities at Different Culture Stages with and without Apex Predator Disturbance

**Figure 1:**
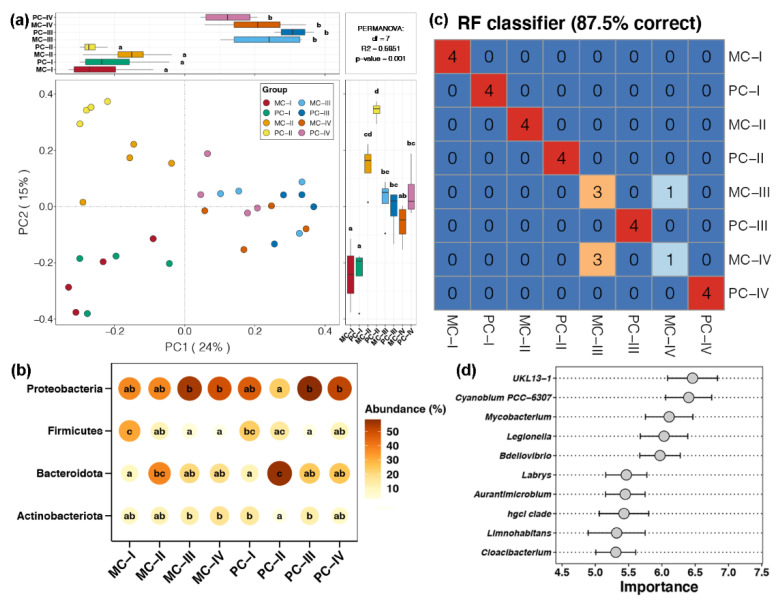
Variation in bacterioplankton communities with culture stage and apex predator disturbance. (**a**) Principal coordinate analysis (PCoA) and PERMANOVA of bacterioplankton communities among different culture stages with and without apex predators. Boxplots (top and right) represent sample distributions from different groups on PC1 and PC2 axes, respectively. Different lowercase letters above each box in the same sub-figure represent significant differences between groups (Tukey’s HSD test, *p* < 0.05). (**b**) Differences in the relative abundance of dominant bacterial phyla among different groups. The size of points represents the relative abundance of different bacterial phyla, and different lowercase letters on the internal side of points from the same row represent significant differences between groups (Tukey’s HSD test, *p* < 0.05). (**c**) Accuracy of the random forest model for distinguishing sample groups. Numbers on the diagonal represent the number of samples in which the results predicted by the random forest model are consistent with the actual results. Numbers in other cells represent the number of samples in which the name of the row is their actual region, but the random forest model predicts them as belonging to the region indicated by the name of the column. (**d**) Importance of key genera identified by the random forest model in distinguish sample groups.

PCoA based on the Bray–Curtis distance showed that bacterioplankton communities at different culture stages clustered separately (Figure 1a). Samples from stage I–II and III–IV were significantly distinguished by PC1 (Tukey’s HSD test, *p* < 0.05), which explained 24% of the total variation. Meanwhile, samples from stage I, III–IV, and II were respectively located in the upper, middle, and lower regions of PC2, which explained 15% of the total variation. Two-way PEMANOVA revealed that the culture stage, apex predator disturbance, and their interaction all significantly impacted bacterioplankton communities (*p* < 0.05, Table S2). Among them, the effect of the culture stage was more powerful than that of apex predator disturbances (0.4234 vs. 0.0675, Table S2). Moreover, the PERMANOVA of bacterioplankton in each culture stage showed that the impact of apex predator disturbances was significant in stage II and III (*p* < 0.05, Table S3). These findings suggested that disturbances by apex predators had less impact than the culture stage, but it had a clear effect in the middle of the culture process.

The relative abundance of bacteria at the phylum level is shown in Figure 1b. *Proteobacteria* (44.83%) was the most dominant phylum, followed by *Bacteroidota* (25.24%), *Firmicutes* (12.41%), and *Actinobacteriota* (11.02%). As culturing progressed, the relative abundance of *Proteobacteria* first decreased in stage II then increased in stage III and IV. Disturbance by apex predators enhanced the reduction in *Proteobacteria* in stage II (Turkey’s HSD test, *p* < 0.05). The relative abundance of *Bacteroidota* during culturing presented the opposite trend to that of *Proteobacteria*, and disturbances by apex predators also enhanced the increase in *Bacteroidota* in stage II (Turkey’s HSD test, *p* < 0.05). For *Firmicutes*, the relative abundance declined continuously with the progression of aquaculture, and disturbances by apex predators had an inhibitory effect on the decrease in *Firmicutes*. The relative abundance of *Actinobacteria* was relatively stable throughout aquaculture, but apex predators decreased the abundance in stage II and IV. The above results show that disturbances by apex predators did not significantly alter bacterioplankton dynamics during the culture process, but it did affect the magnitude of changes in bacterial abundances.

Using the random forest model, we determined that the composition of bacterioplankton communities was strongly linked to the culture stage and apex predator disturbances (Figure 1c). Samples could be assigned to different groups with 87.5% accuracy, except for samples from stage III and IV of MC aquaculture ponds. Bacterial genera most important for sample discrimination were *UKL13-1*, *Cyanoblum PCC-6307*, *Mycobacterium*, *Legionella*, *Bdellovibrio*, *Labrys*, *Aurantimicrobium*, *hgcl clade*, *Limnohabitans*, and *Cloacibacterium* (Figure 1d). Among these key genera, most were present in relatively low abundance (Figure S2). Combining the results of changes in overall bacterioplankton communities with those of random forest model analysis revealed that apex predators significantly altered the prevalence of low-abundance species, but they did not dramatically alter overall community composition.

### 2.3. Environmental Adaptation of Bacterioplankton Communities

Eight environmental factors were measured to assess the effects of the culture stage and apex predators (Figure S3). The water temperature was significantly higher and lower in stage I and IV, respectively, compared with other culture stages (Tukey’s HSD test, *p* < 0.05, Table S4), but no significant difference was observed between MC and PC ponds at the same culture stage (Tukey’s HSD test, *p* > 0.05, Figure S3a). Water pH in stage I was significantly higher in PC ponds than in MC ponds (Tukey’s HSD test, *p* < 0.05, Table S4), but there were no differences in other culture stages (Tukey’s HSD test, *p* > 0.05, Figure S3b). The DO content in PC ponds was significantly lower than in MC ponds in stage I (Tukey’s HSD test, *p* < 0.05, Table S4), but it gradually increased to a similar level as that of MC ponds with aquaculture progression (Figure S3c). Concentrations of ammonia and nitrite in cultural ponds first increased then decreased with aquaculture progression (Figure S3d,e). Disturbance by apex predators suppressed the changes in ammonia and nitrite concentrations to some extent (Table S4). Concentrations of nitrate and TN presented the opposite trend to ammonia and nitrite during aquaculture progression (Figure S3f,g), and disturbances by apex predators enhanced the variation in nitrate concentration, but had no obvious effect on TN (Table S4). Levels of TP were not significantly altered at different culture stages with or without apex predator disturbances (Tukey’s HSD test, *p* > 0.05, Figure S3h).

**Figure 2:**
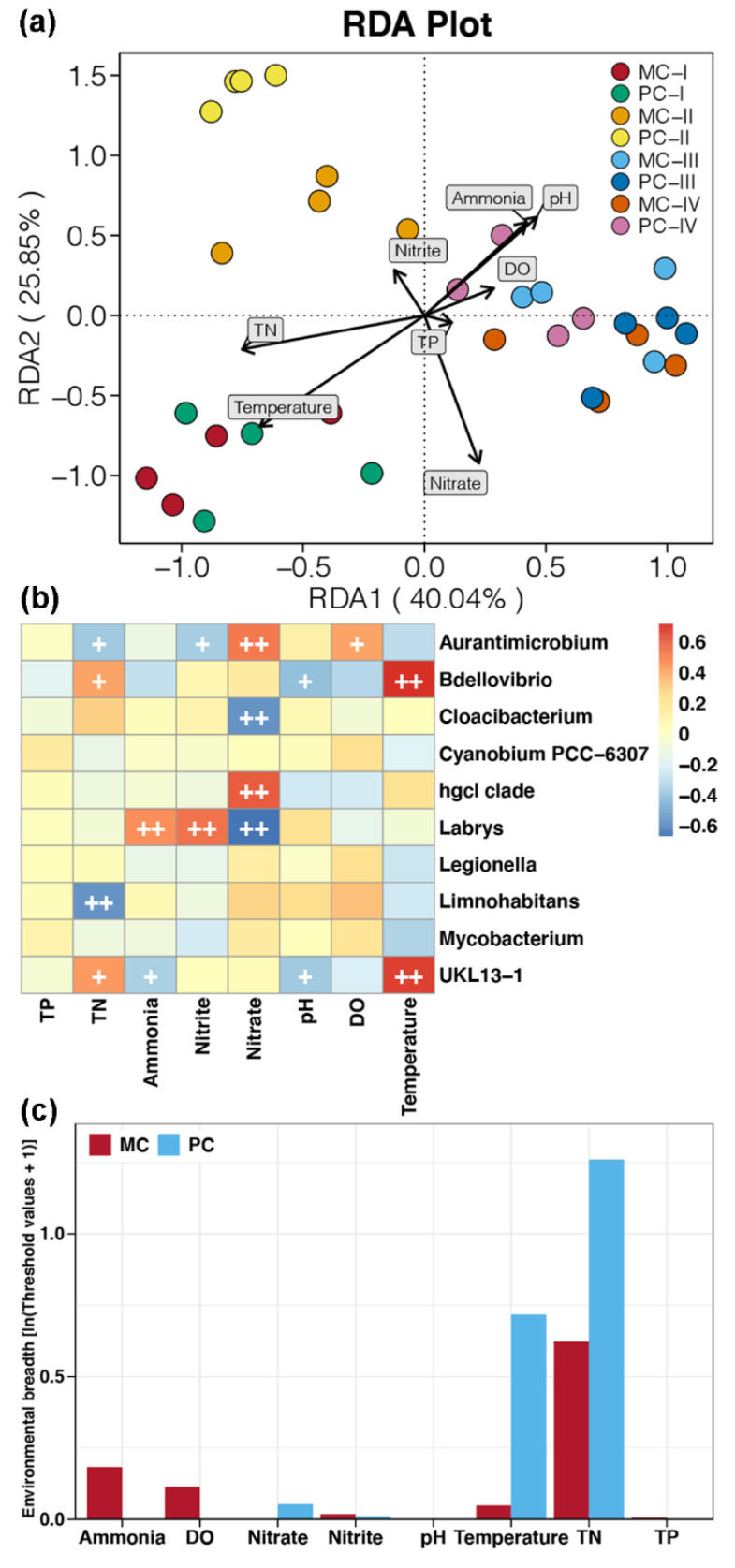
Environmental adaptation of bacterioplankton communities. (**a**) Redundancy analysis evaluating the relationship between bacterioplankton communities and environmental factors during aquaculture progression in MC and PC groups. (**b**) Heat map revealing significant correlations between the relative abundances of key genera obtained by the random forest model and environmental factors. The Spearman correlation coefficient is shown by the color of each heatmap cell. A significant correlation was confirmed if the *p*-value with Bonferroni adjustment was <0.05 (+) or 0.01 (++). (**c**) Environmental breadth of bacterial communities, estimated by threshold values of MC and PC bacterioplankton taxa in response to environmental factors, calculated using TITAN. DO, dissolved oxygen; TN, total nitrogen; TP, total phosphorus.

Based on RDA, 65.89% of the variance in bacterioplankton communities in MC and PC ponds could be explained by selected environmental factors (Figure 2a). Significant correlations were found between most environmental factors and bacterioplankton communities, except for DO, nitrite, and TP (Table S5). Bacterioplankton communities from different culture stages were clearly distinguished by environmental factors. Water temperature was positively correlated with bacterioplankton communities from stage I, while the effects of ammonia and pH were diametric. In addition, concentrations of nitrate and TN were negatively correlated with bacterioplankton communities from stage II and III–IV, respectively.

In addition, correlations between the relative abundances of key genera and environmental factors were investigated to distinguish bacterioplankton communities (Figure 2b). Water temperature and pH were positively and negatively correlated with the relative abundances of *UKL13-1* and *Bdellovibrio*, respectively (Spearman’s correlation, *p* < 0.05). Nitrate concentrations were positively correlated with the relative abundances of *hgcl clade* and *Aurantimicrobium*, but negatively correlated with that of *Labrys* and *Cloacibacterium* (Spearman’s correlation, *p* < 0.05). Levels of ammonia and nitrite were positively correlated with the relative abundance of *Labrys*, but negatively correlated with that of *UKL13-1* and *Aurantimicrobium*, respectively (Spearman’s correlation, *p* < 0.05). In addition, the TN concentration was positively correlated with the relative abundances of *UKL13-2* and *Bdellovibrio*, but negatively correlated with those of *Limnohabitans* and *Aurantimicrobium* (Spearman’s correlation, *p* < 0.05). Moreover, the relative abundances of *UKL13-1* and *Bdellovibrio* were negatively correlated with the water pH (Spearman’s correlation, *p* < 0.05).

Furthermore, environmental breadth was used to investigate bacterioplankton responses to environmental factors. Bacterioplankton communities in MC ponds exhibited a broader range of environmental breadth for DO, ammonia, nitrite, and TP, while a broader environmental breadth was observed for water temperature, nitrate, and TN in PC samples (Figure 2c). Overall, the environmental breadth of bacterioplankton communities in PC ponds was broader than that in MC ponds. This result suggests a stronger environmental adaptation for bacterioplankton communities in aquaculture ponds with apex predator disturbance.

### 2.4. Community Assembly of Bacterioplankton Affected by Apex Predators

**Figure 3:**
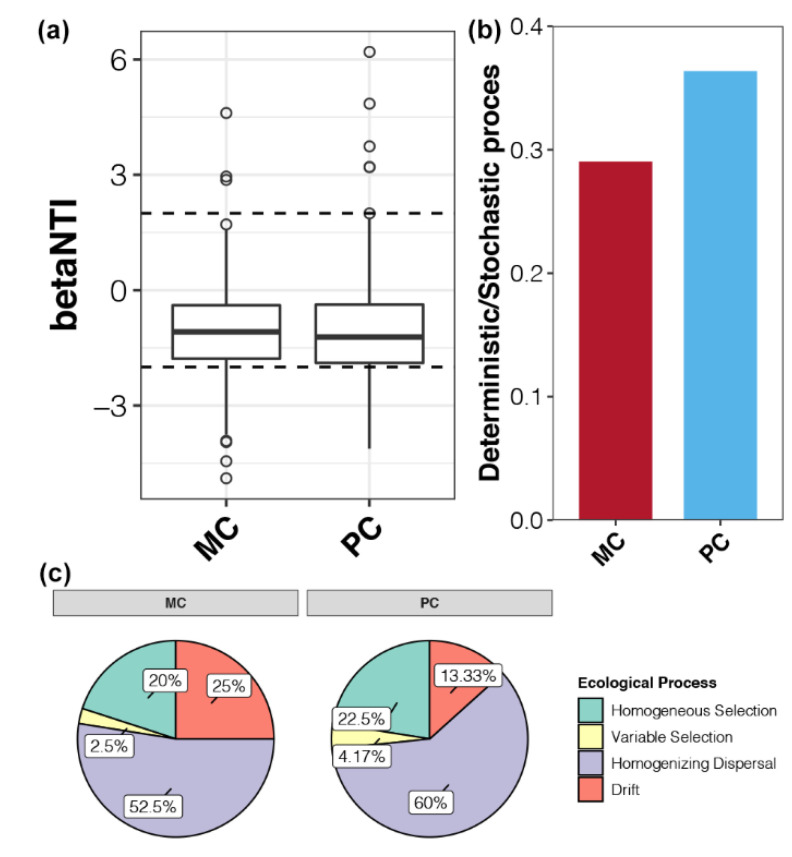
Community assembly of bacterioplankton is affected by apex predators. (**a**) betaNTI values for bacterioplankton communities for MC and PC ponds. Horizontal dashed lines indicate betaNTI significance thresholds of +2 and −2. (**b**) Ratio of relative contributions of deterministic and stochastic processes for the assembly of bacterioplankton communities in MC and PC ponds. (**c**) Relative contributions of ecological processes to the assembly of bacterioplankton communities in MC and PC ponds.

Based on the null model, the relative contributions of deterministic and stochastic processes on the community assembly of bacterioplankton in MC and PC ponds were estimated. The median values of betaNTI for bacterioplankton communities in MC and PC ponds were between −2 and 2, indicating that stochastic processes were dominant for community assembly (Figure 3a). In contrast with MC samples, deterministic processes contributed more to the assembly of bacterioplankton communities in PC ponds (Figure 3b). Homogenizing dispersal, a stochastic process, was the dominant ecological process shaping bacterioplankton communities in both MC and PC ponds, with corresponding relative contributions of 52.5% and 60.0%, respectively (Figure 3c). Additionally, the relative contribution of drift was significantly lower in PC samples (13.33%) than in MC ponds (25.0%; Figure 3c). For deterministic processes, the contribution of homogeneous selection was stronger than that of variable selection in both MC and PC ponds, and both were slightly higher in PC ponds than MC samples (Figure 3c). These findings indicated that apex predator disturbances enhanced the relative contributions of deterministic processes for bacterioplankton community assembly by improving environmental filtering and reducing drift.

### 2.5. Ecological Function of Bacterioplankton Affected by Apex Predators

**Figure 4:**
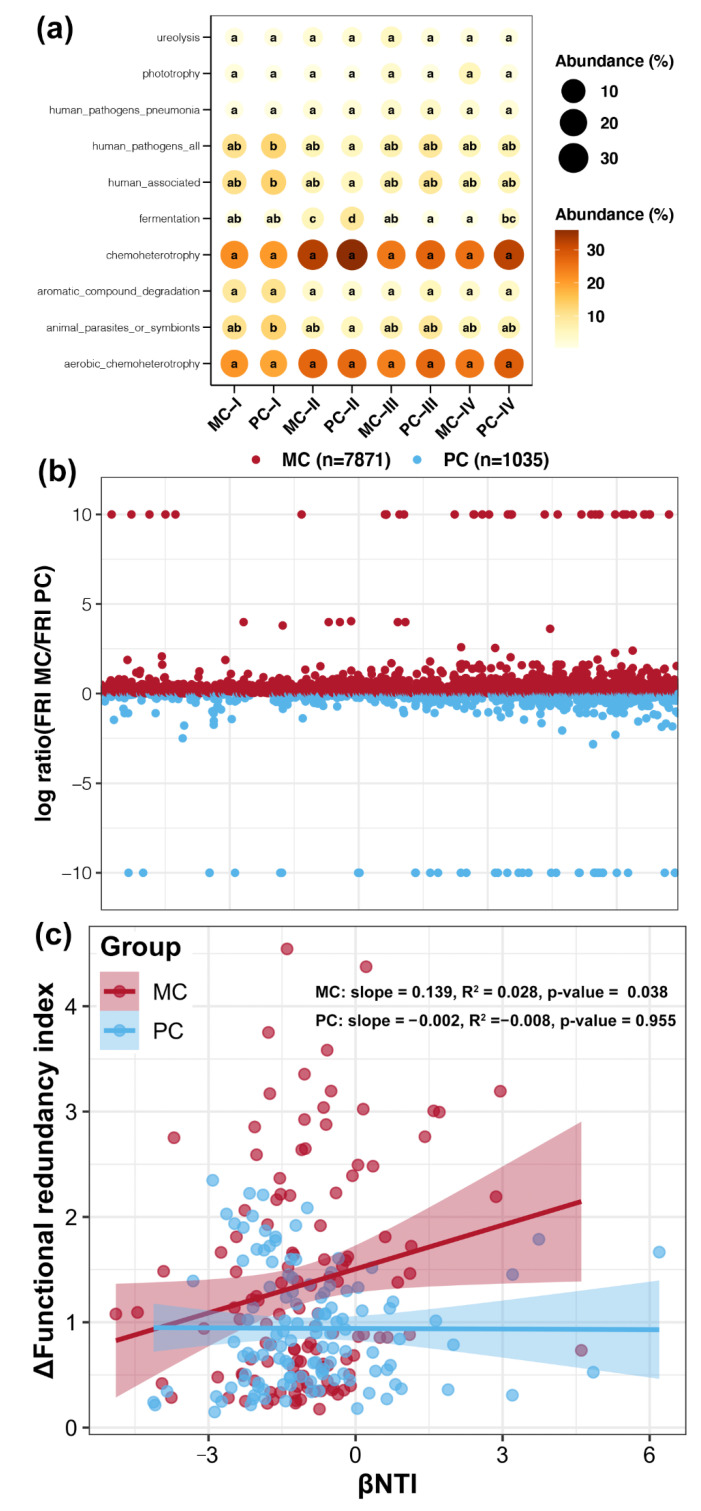
Ecological functions of bacterioplankton communities affected by apex predators. (**a**) Differences in relative abundances of dominant bacterial phyla among different groups. The size of points represents the relative abundance of different bacterial phyla, and different lowercase letters on the internal side of points from the same row represent significant differences between groups (Tukey’s HSD test, *p* < 0.05). (**b**) Ratio of functional redundancy indices (FRI) between bacterioplankton communities in MC and PC ponds. A log ratio > 0 denotes that a function is more redundant in MC ponds, while <0 denotes more redundancy in PC ponds. (**c**) Linear regression between betaNTI and FRI for bacterioplankton communities in MC and PC ponds.

Functional profiles of bacterioplankton communities were predicted using FAPROTAX software, and significant differences among all groups were determined. Functions related to chemoheterotrophy were dominant in bacterioplankton communities in both MC and PC ponds, followed by several functions associated with humans or other animals (Figure 4a). Importantly, disturbances by apex predators significantly reduced the relative abundances of functional terms related to humans and other animals (Tukey’s HSD test, *p* < 0.05, Figure 4a). Furthermore, significantly elevated fermentation functions were found in bacterioplankton communities at stage II compared with other stages, and disturbances by apex predators enhanced this increase (Tukey’s HSD test, *p* < 0.05, Figure 4a).

Based on the KEGG orthology level 3 functional profiling results obtained by TAX4Fun2 prediction, 7871 functions showed higher redundancy in bacterioplankton communities of MC ponds, whereas 1035 functions showed higher redundancy in PC bacterioplankton communities (Figure 4b). This suggested a decrease in functional redundancy in the bacterioplankton of aquaculture ponds with apex predator disturbances. Additionally, betaNTI was significantly positively correlated with changes in FRI for bacterioplankton in MC ponds (*p* < 0.05), while there was no obvious correlation in PC ponds (*p* > 0.05, Figure 4c). This implied that ecological community processes had weak effects on the functions of bacterioplankton in aquaculture ponds with apex predators.

### 2.6. Community Stability of Bacterioplankton Affected by Apex Predators

**Figure 5:**
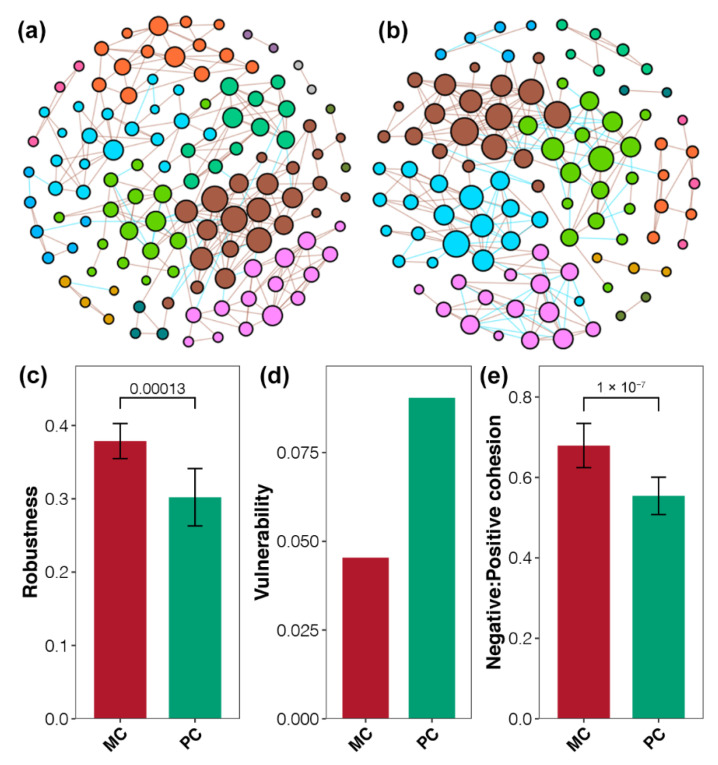
Community stability of bacterioplankton communities is affected by apex predators. (**a**,**b**) Molecular ecological networks of bacterioplankton communities in MC and PC ponds based on RMT analysis of OTU profiles. Modules are labeled in different colors in the respective networks. (**c**–**e**) Differences in robustness, vulnerability, and negative:positive cohesion between bacterioplankton communities in MC and PC ponds. Values on bars are *p*-values from Student’s *t*-tests between MC and PC networks.

To investigate bacterioplankton patterns in aquaculture ponds, MENs were constructed for MC and PC samples (Figure 5a,b). The MEN for MC ponds had 119 nodes and 295 edges with an average degree of 4.958, and the diameter and density were 13 and 0.042, respectively (Table S6). The MEN for PC ponds had 98 nodes and 256 edges with an average degree of 5.224, and the diameter and density were 13 and 0.054, respectively (Table S6). Differences in topological properties between these two MENs indicated a slight correlation with bacterioplankton in PC ponds.

Robustness, vulnerability, and cohesion indices were calculated to evaluate the community stability of bacterioplankton in aquaculture ponds. Significantly higher robustness was found in in MEN for MC ponds compared with that for PC ponds (Student’s *t*-test, *p* < 0.05, Figure 5c). Network vulnerability confirmed that the MEN for MC ponds was more stable than that for PC ponds (Figure 5d). Moreover, N:P cohesion was significantly higher in the MEN for MC ponds compared with that for PC ponds (Student’s *t*-test, *p* < 0.05, Figure 5e). Overall, all indices demonstrated that MENs for bacterioplankton had higher stability in aquaculture ponds without apex predator disturbance.

### 2.7. Mechanisms of Apex Predators Shaping the Bacterioplankton

**Figure 6:**
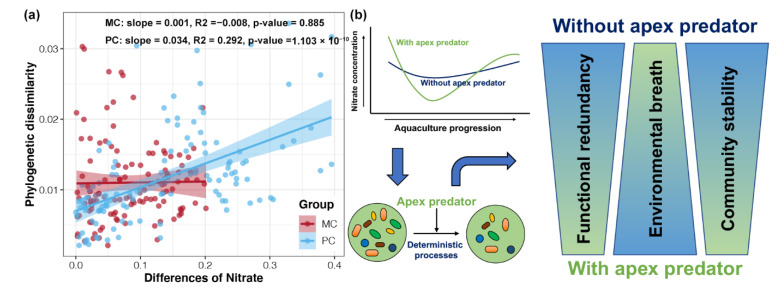
Mechanisms of apex predators’ shaping of bacterioplankton communities. (**a**) Linear regression analysis of differences in nitrate concentrations and phylogenetic dissimilarities for bacterioplankton in MC and PC ponds. (**b**) Conceptual models revealing the mechanism of bacterioplankton variation in aquaculture ponds affected by apex predator disturbance.

To disentangle the key environmental factors associated with apex predator disturbances that influence the community assembly of bacterioplankton, relationships between environmental factors and phylogenetic distances of bacterioplankton were determined for MC and PC ponds. Significant correlations were observed for water temperature, pH, ammonia, and TN with the betaMNTD of bacterioplankton in MC ponds (*p* < 0.05, Table S7). Meanwhile, water temperature, ammonia, and nitrate were found to be significantly correlated with the betaMNTD of bacterioplankton in PC ponds (*p* < 0.05, Table S7). Among them, only nitrate concentrations were significantly correlated with the phylogenetic dissimilarity of bacterioplankton in PC ponds, but not in MC ponds (Figure 6a).

Based on our data, we constructed a conceptual framework to describe the assembly processes for bacterioplankton communities in aquaculture ponds with and without apex predators (Figure 6b). With aquaculture progression, the nitrate concentration in ponds first decreased, then returned to initial levels. Disturbance by apex predators amplified the variation in nitrate concentration, which led to an increased contribution of deterministic processes to bacterioplankton community assembly. Ultimately, bacterioplankton in culture ponds with apex predators exhibited higher environmental adaptation but lower functional redundancy and community stability.

## 3. Discussion

### 3.1. Dynamics of Bacterioplankton Communities during the Crustacean Aquaculture

Significant variations in the diversity and composition of bacterial communities during the culture process have been reported for many aquaculture systems [6,25,26]. In the present study, the dynamics of bacterioplankton communities in crustacean aquaculture ponds during the whole process were investigated. However, no differences in alpha diversity were observed (Figure S1). Similar results were observed in our previous study on an in-pond raceway’s recirculating culture system [1]. Both studies were conducted in freshwater aquaculture systems under strictly human control, and aquaculture activities were of a relatively short duration. The fixed species pool and relatively stable climatic conditions could explain the small changes in bacterial diversity. In contrast, the composition of bacterioplankton communities was significantly altered during the aquaculture process, as observed in most similar studies. A stable alpha diversity, but with differences in community composition, indicated that the abundances of major bacterial taxa in crustacean aquaculture systems were altered during the culture process.

Bacteroidota species were more abundant in the bacterioplankton community of crustacean aquaculture ponds at stage II compared with other culture stages (Figure 1b). Bacteroidota plays a role in organic substance circulation by mineralizing a wide variety of organic compounds [27]. The sequencing of Bacteroidota genomes confirmed the presence of numerous carbohydrate-active enzymes, which can improve the degradation of water-borne organic compounds [28]. In addition, the relative abundance of Firmicutes decreased in the middle of culture progression (Figure 1b). Firmicutes are known to function in the metabolism of fat, and they are correlated with the body fat content of animals [29]. These findings suggest high organic matter degradation activity and a low capacity for fat production in the middle stages of crustacean aquaculture.

### 3.2. Effects of Apex Predator Disturbance on Bacterioplankton Communities of Crustacean Aquaculture Systems

Although variation in bacterioplankton related to the culture stage was stronger than variation related to apex predator disturbance, it also affected bacterioplankton communities by enhancing or suppressing specific changes. The increase in Bacteroidota in stage II was enhanced by apex predator disturbances (Figure 1b). Meanwhile, functions associated with fermentation were more abundant in stage II of cultural ponds with apex predator disturbances (Figure 4a). These results indicated that apex predators further strengthened the degradation of organic matter and weakened fat production in the middle stages of crustacean aquaculture. In addition, functions of bacterioplankton communities related to humans and other animals were significantly decreased by apex predator disturbances (Figure 4a). Among them, a considerable number of bacteria were associated with pathogenicity. They could originate from human manipulation during the culture process and/or eutrophication caused by the accumulation of feces, waste, and dead animals in culture ponds [30,31].

Mandarin fish (*S. chuatsi*), the apex predator investigated in this study, is a carnivorous fish that can consume old, sick, and almost dying animals and excessive culturing resources in aquaculture ponds. Aquacultures with apex predators may reduce foreign bacteria while suppressing the propagation of harmful bacteria [5]. In the present work, the increase in ammonia at stage III was inhibited, but the decrease of nitrate at stage II was promoted by apex predator disturbances (Figure S3). Excess ammonia is a toxic substance for culture species in aquaculture ponds, and nitrate is a key eutrophication indicator [32,33]. Based on these results, apex predators may support the survival of culture species by suppressing toxic substances and eutrophication.

### 3.3. Stochastic Processes Dominate Bacterioplankton Community Assembly in Crustacean Aquaculture Systems

Our results revealed that stochastic processes dominated the assembly of bacterioplankton communities in crustacean aquaculture systems (Figure 3). Similar results were also reported for bacterial community assembly in diverse natural and artificial ecosystems [1,34,35]. However, studies have confirmed that deterministic processes dominate microbial community assembly, including bacterioplankton, in a highly urbanized estuarine ecosystem [17] and in soil bacteria communities in rice fields receiving long-term fertilization [23]. These results imply that crustacean aquaculture systems might not be strong enough to alter environmental conditions and select for specific microorganisms. In addition, homogenizing dispersal was found to be the strongest stochastic process shaping bacterioplankton communities in this study, whereas dispersal limitation did not appear to contribute (Figure 3c). Our system was enclosed aquaculture ponds, in which no factors limit the dispersal of bacterioplankton. Powerful hydrological mixing can increase homogenizing dispersal and ecological drift, making stochastic processes more prominent [36].

### 3.4. Mechanisms Underlying the Bacterioplankton Community Assembly Affected by Apex Predators

Our results showed that apex predator disturbances slightly increased the contribution of deterministic processes in shaping the bacterioplankton community of crustacean aquaculture systems (Figure 3b). This finding is in agreement with the relatively limited variation in bacterioplankton community composition between MC and PC samples at the same cultural stage (Figure 1a). Even with small differences in community composition, we found that apex predator disturbances significantly altered other ecological properties of the bacterioplankton community in crustacean aquaculture ponds. First, bacterioplankton in PC ponds exhibited a broader response threshold to most environmental factors compared with the community in MC ponds (Figure 2c). This discrepancy may be due to greater changes in environmental factors during aquaculture activities in the presence of apex predators (Figure S3). This phenomenon makes it necessary for surviving bacteria to be able to adapt to a broad range of environmental conditions [37]. Second, apex predator disturbances reduced the functional redundancy of bacterioplankton communities (Figure 4b). Before aquaculture activities, bacterioplankton communities in cultural ponds originate from the natural environment, typically characterized by high species connectivity and functional redundancy [38]. Greater changes in environmental factors under apex predator disturbances could indirectly reduce some community members that share similar ecological characteristics by removing microorganisms that cannot adapt [39], resulting in a reduction in functional redundancy. The higher functional redundancy of a microbial community implies that it can better resist strong external stresses and achieve better stability under environmental changes [11]. Therefore, the reduced functional redundancy could be linked to a less stable bacterioplankton community under apex predator disturbances (Figure 5).

Conceptual frameworks for key environmental factors affecting community assembly mechanisms have been proposed for some specific environments. For example, the soil pH mediates bacterial community assembly during soil succession, since extreme pH leads to deterministic assembly, and neutral conditions result in strong stochastic processes [22]. Available potassium is decisive in determining the balance between the stochasticity and determinism of rhizosphere and the bulk soils in rice cropping ecosystems, resulting in poor environmental adaptation and a high stability of bacterial communities in the rhizosphere [23]. Moreover, DO mediates the dynamics of ecological processes in bacterioplankton community assembly of eutrophic lakes, inducing stronger environmental adaptation of rare bacterial taxa [24]. In the present study, we observed that changes in nitrates were correlated with the phylogenetic distance of bacterioplankton communities in crustacean aquaculture systems with apex predator disturbance. This suggested that greater changes in nitrates caused by apex predator disturbances could adjust the balance between stochastic and deterministic processes, resulting in enhanced contributions of deterministic processes (Figure 6c). To the best of our knowledge, this is the first study to propose a conceptual framework linking microbial community assembly and underlying ecological causes in aquaculture environments with apex predator disturbance.

## 4. Materials and Methods

### 4.1. Aquaculture Progression and Sample Collection

The experiment was conducted at a local aquaculture farm in Changzhou City, Jiangsu Province, China (120°8′4.3008″ E, 31°41′14.3772″ N). Eight aquaculture ponds containing Chinese mitten carb (*Eriocheir sinensis*) and oriental river prawn (*Macrobrachium nipponensis*) were studied in this work, of which four ponds also contained mandarin fish (*Siniperca chuatsi*) as apex predators (PC group), while the other four ponds remained without apex predators (MC group). The area of each aquaculture pond was ~10 mu. Intact and healthy crabs (~7.50 g) were stocked in each aquaculture pond at a density of 1000 crabs mu^−1^. Prawns (~8.30 g) were ovigerous and stocked in each aquaculture pond at a density of 0.20 kg mu^−1^. The mandarin fish added in the PC group were ~5 cm in length and stocked at a density of 10 individuals mu^−1^. Experimental diets were commercial compound feed and frozen trash fish. Crabs were fed at 17:00 once every day, and the daily feeding ration was 3% of crab body weight. The water depth of aquaculture ponds was kept at ~1.2 m throughout the experimental period (water was added accordingly). In addition, 20% of the water in each aquaculture pond was exchanged once a month. All farming operations were strictly consistent, and the experimental period was from 7 August to 27 September.

During aquaculture progression, five water samples (~5 L) were collected from each pond at all four stages (7 August for stage I, 28 August for stage II, 14 September for stage III, and 27 September for stage IV) using a horizontal Van Dorn Water Sampler (KC Denmark A/S, Silkeborg, Denmark) and mixed according to the five-point sampling method. All samples were immediately placed in sterilized containers in a portable ice box and transported to the laboratory within 8 h of collection. Approximately 2 L of each sample was used to concentrate bacterial cells using a water filtration apparatus with a 0.22 μm Durapore Membrane Filter (Millipore, Burlington, MA, USA). Filter membranes were stored at −80 °C for further DNA extraction, and the remaining water samples were used to measure environmental factors.

### 4.2. Measurement of Environmental Factors

The pH, dissolved oxygen (DO), and water temperature were measured using an HQ4300 Multi/ISE/3-Channel HQ Series Portable Meter (Hach, Loveland, CO, USA). Total phosphorus (TP) and total nitrogen (TN) concentrations in water samples were determined as described by Qian and Fu (1987) [40]. Ammonia, nitrite, and nitrate in water samples were determined as described by Laskov et al. (2007) and Tu et al. (2010) [41,42].

### 4.3. DNA Extraction and 16S rRNA Sequencing

Bacterial DNA in water samples was extracted using a PowerWater DNA Isolation Kit (Mobio, Carlsbad, CA, USA) following the manufacturer’s instructions. Agarose gel electrophoresis (1%) was used to confirm the successful extraction of DNA. The concentration and purity of extracted DNA were measured by a NanoPhotometer Classic (Implen, Munich, Germany). Samples were stored at −20 °C for further analysis.

The V4–V5 region of the bacterial 16S ribosomal RNA gene was amplified by PCR (95 °C for 2 min, followed by 25 cycles at 95 °C for 30 s, 55 °C for 30 s, and 72 °C for 30 s, and a final extension at 72 °C for 5 min) using primers 515F (5′-GTGCCAGCMGCCGCGG-3′) and 907R (5′-CCGTCAATTCMTTTRAGTTT-3′). Each PCR amplification was performed in triplicate in 20 μL mixtures containing 4 μL of 5× FastPfu Buffer, 2 μL of 2.5 mM dNTPs, 0.8 μL of each primer (5 μM), 0.4 μL of FastPfu Polymerase, and 10 ng of template DNA. Amplicons were extracted from 2% agarose gels and purified using an AxyPrep DNA Gel Extraction Kit (Axygen Biosciences, Union City, CA, USA) according to the manufacturer’s instructions. Purified PCR products were quantified by Qubit 3.0 (Life Invitrogen) and mixed equally. Amplicon libraries were generated using a TruSeq Nano DNA LT Library Prep Kit (Illumina, San Diego, CA, USA) according to the manufacturer’s instructions. Library quality was assessed using a QuantiFluor dsDNA system (Promega, Madison, WI, USA) and an Agilent Bioanalyzer 2100 system (Agilent, Santa Clara, CA, USA). Finally, libraries were sequenced using an Illumina Novaseq6000 platform with a 250 bp paired-end strategy at BIOZERON Biotech. Co., Ltd. (Shanghai, China).

### 4.4. Sequence Data Processing

Quality filtering of raw reads was conducted using QIIME (v.1.9.1, Caporaso et al, Flagstaff, AZ, USA) [43] to generate high-quality clean tags. Reads with average Phred scores < 20, that contained ambiguous bases or had homopolymer runs that exceeded eight, had mismatches in primers, and had sequence lengths < 250 bp were removed [44]. FLASH was applied to assemble paired end reads that overlapped by >10 bp without any mismatches into tags [45]. These tags were then appointed to samples based on their unique barcodes and truncated by removing barcodes and primer sequences. Chimeras were also distinguished and eliminated using QIIME v 1.9.1. Sequences with 97% similarity were assigned to the same operational taxonomic unit (OTU) by UCLUST with the open reference strategy [46]. Representative sequences of each OTU were screened using the default method, and taxonomically assigned using the SILVA database (Release 138) [47]. An OTU abundance table was constructed based on the number of tags that belonged to each OTU in each sample. Singletons (abundance < 0.001%) were then removed to improve the efficiency of data analysis [44]. Finally, OTU abundance data were normalized using a standard number of tags according to the sample with the lowest number of tags (16,984 tags). All subsequent analyses were performed using normalized OTU abundance data.

### 4.5. Statistical Analysis

Four alpha diversity indices of bacterioplankton communities, namely, Chao1, Shannon, Peilou_J, and Pd_faith, were calculated to estimate richness, diversity, evenness, and evolution, respectively. Ecologically relevant functions of bacterioplankton communities were established using FAPROTAX software [48]. Differences in environmental factors, alpha diversity indices, and the relative abundances of dominant bacterial phyla and functional terms among different groups were confirmed using Tukey’s honestly significant difference (HSD) test. To evaluate variations in bacterioplankton communities, Bray–Curtis distances among different samples were calculated. Principal coordinate analysis (PCoA) and PERMANOVA tests based on Bray–Curtis distances were performed to assess the effects of the culture stage and apex predator disturbances on bacterioplankton community compositions. In addition, random forest analysis was performed to assign bacterioplankton communities into groups. The accuracy of the random forest model was displayed as a heat map, and key bacterial genera for sample discrimination were identified.

Redundancy analysis (RDA) was conducted to determine the contributions of environmental factors to shifts in bacterioplankton communities. Spearman’s correlations were estimated to assess relationships between key genera for sample discrimination and environmental factors, and the results were visualized by heat map. To evaluate environmental adaptation, the environmental breadth of bacterioplankton communities in MC and PC ponds was estimated [23]. Briefly, threshold indicator taxa analysis (TITAN) was conducted, and the environmental breath was determined by calculating the sum of taxa scores for OTUs for each environmental factor [49]. A higher value of environmental breadth of the bacterioplankton community for a specific environmental factor indicated that the community had higher environmental adaptation.

Null model analysis was performed to quantify the relative contributions of deterministic and stochastic processes on bacterioplankton community assembly [16]. Specifically, ecological processes governing bacterial community assembly were identified based on the β-nearest taxon index (βNTI) and the Raup–Crick metric (RC). The fraction of pairwise comparisons with βNTI < −2 was considered the percentage of homogeneous selection, whereas those with βNTI > 2 was considered the percentage of variable selection. Next, the RC taxonomic diversity metric was used to partition the remaining pairwise comparisons with |βNTI| ≤ 2. The fraction of pairwise comparisons with RC < −0.95 was treated as the percentage of homogenizing dispersal, while those with RC > 0.95 were treated as the dispersal limitation. The remaining comparisons with |βNTI| ≤ 2 and |RC| ≤ 0.95 represented the drift percentages. Among these ecological processes, homogeneous and variable selection are deterministic processes, and homogenizing dispersal, dispersal limitation, and drift are stochastic processes.

The beta mean nearest taxon distance (betaMNTD) of bacterioplankton communities was calculated to evaluate phylogenetic dissimilarities among different samples. Differences in each of the environmental factors between any two samples were calculated, and linear regression analysis was performed on changes in environmental factors with phylogenetic dissimilarities for bacterioplankton communities in MC and PC ponds based on ordinary least-squares regression. The functional redundancy index (FRI) of each sample was determined based on the similarity of 16S rRNA gene sequences using the Tax4Fun2 method [24]. FRI refers to the proportion of species capable of possessing a particular Kyoto Encyclopedia of Genes and Genomes (KEGG) function and their phylogenetic relationship to each other [50]. Linear regression analysis was performed on the changes in FRI and βNTI in MC and PC ponds based on ordinary least-squares regression.

To further decipher differences in bacterioplankton distribution patterns affected by apex predator disturbance, MEN analysis was performed using the random-matrix-theory-based approach and high-throughput sequencing data, according to the MEN analysis pipeline [14]. This analysis refers to previous research [51], and network graphs were visualized using the Gephi interactive platform [52]. The robustness, vulnerability and cohesion of MENs for MC and PC samples were calculated to evaluate the stability of bacterioplankton communities according to a previous study [53].

All analyses were completed using base, multcomp, vegan, randomForest, picante, pheatmap, TITAN2, Tax4Fun2, and ggplot2 packages in the R (v.4.0.3, R Core Team, Vienna, Austria) platform.

## 5. Conclusions

In conclusion, significant variations in the composition and functions of bacterioplankton communities were observed in crustacean aquaculture ponds at different culture stages. In the middle stages of aquaculture progression, bacteria involved in organic matter degradation were increased, and bacteria involved in pathogenicity were decreased by the apex predator. Moreover, greater changes in environmental factors were also observed in aquaculture ponds with apex predators, and the mandarin fish effectively maintains a good culturing environment and supports the survival of culture species by suppressing toxic substances and eutrophication. Stochastic processes dominated the assembly of bacterioplankton communities during aquaculture, but apex predator disturbances increased the contribution of deterministic processes. Nitrate had a decisive role in mediating the balance between stochastic and deterministic processes in bacterioplankton community assembly under apex predator disturbance. Variations in bacterioplankton communities with apex predator disturbances resulted in broader environmental adaptation but less functional redundancy and community stability. These findings provided a greater understanding of bacterioplankton community assembly in aquaculture systems affected by apex predator species.

## Data Availability

The datasets presented in this study can be found in online repositories. The names of the repository/repositories and accession number(s) are PRJNA847361.

## Supplementary Materials

The following supporting information can be downloaded at: https://www.mdpi.com/article/10.3390/ijms231810785/s1.
