# Supplementary material for: Apex Predators Enhance Environmental Adaptation but Reduce Community Stability of Bacterioplankton in Crustacean Aquaculture Ponds
Source: Int J Mol Sci. 2022 Sep 15;23(18):10785. doi: 10.3390/ijms231810785 (PMC9506085; doi:10.3390/ijms231810785)
Supplement: Supplementary file 1 [file ijms-23-10785-s001.zip › ijms-1921619-supplementary.pdf]

Supplemental Material for

Apex predators enhance environmental adaptation but reduce community stability of bacterioplankton in crustacean aquaculture ponds

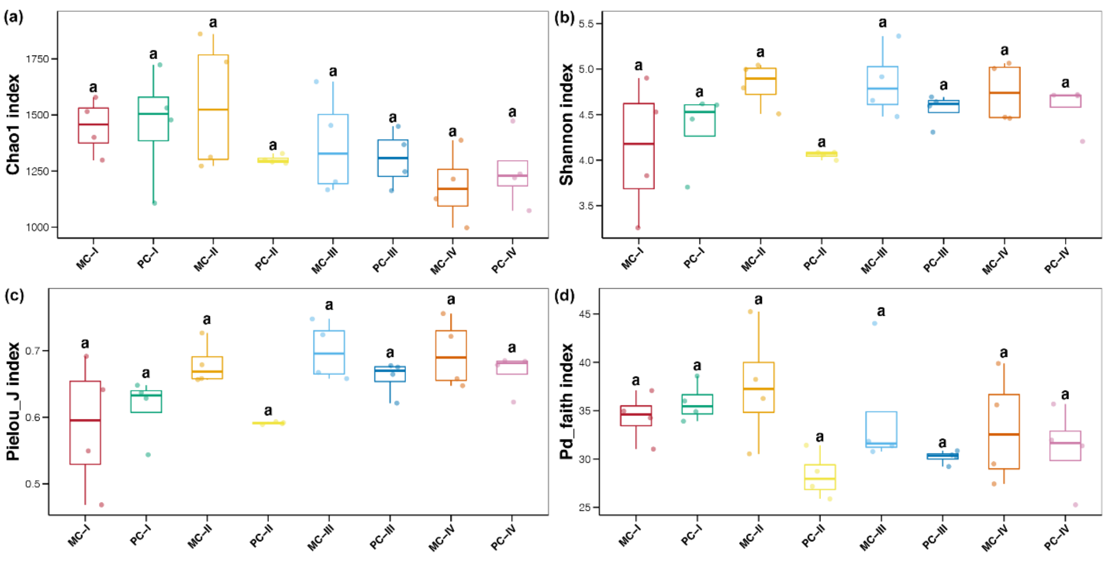

Figure S1. Differences of alpha diversity indices among bacterioplankton communities in crustacean aquaculture ponds with and without apex predator during the whole aquaculture process. Different lowercase letters above each box in the same sub-figure represent significant differences between groups (Tukey's HSD test, $p < 0.05$).

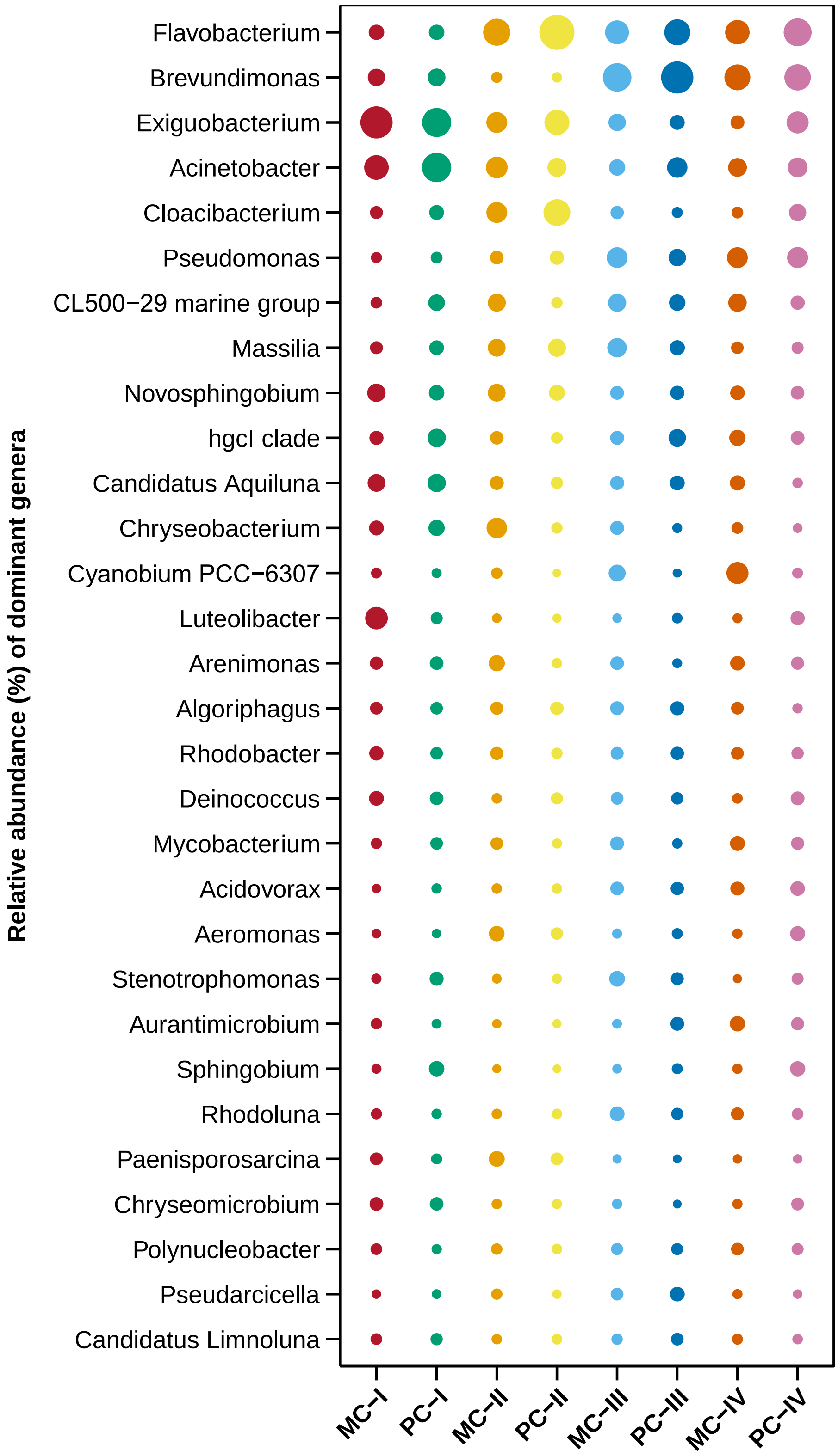

Figure S2. Relative abundance of dominant bacterial genera among different samples.

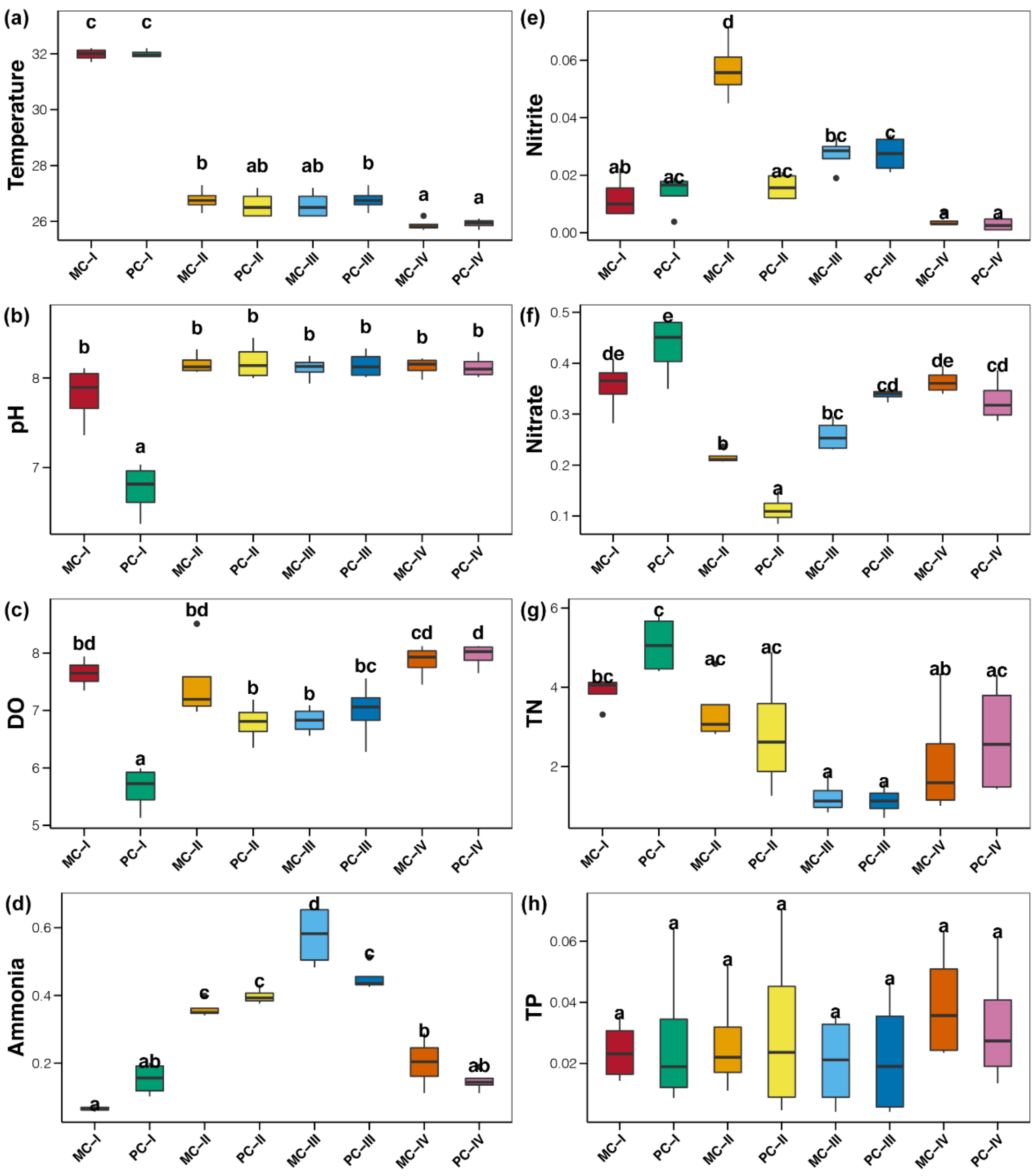

Figure S3. Differences of environmental factors among crustacean aquaculture ponds with and without apex predator during the whole aquaculture process. Different lowercase letters above each box in the same sub-figure represent significant differences between groups (Tukey's HSD test, $p < 0.05$).

Table S1. The p-values of Tukey's HSD tests for alpha diversity indices of bacterioplankton in cultured ponds among different stages with and without the apex predator species.

Group1 - Group2	Chao1	Shannon	Pielou_J	Pd_faith
**PC-I - MC-I**	1.000	0.991	0.993	1.000
**MC-II - MC-I**	0.996	0.186	0.184	0.956
**PC-II - MC-I**	0.952	1.000	1.000	0.501
**MC-III - MC-I**	0.999	0.165	0.062	1.000
**PC-III - MC-I**	0.962	0.737	0.460	0.859
**MC-IV - MC-I**	0.517	0.317	0.077	1.000
**PC-IV - MC-I**	0.820	0.677	0.334	0.954
**MC-II - PC-I**	0.998	0.600	0.568	0.999
**PC-II - PC-I**	0.931	0.955	0.997	0.239
**MC-III - PC-I**	0.997	0.558	0.266	1.000
**PC-III - PC-I**	0.943	0.991	0.888	0.575
**MC-IV - PC-I**	0.466	0.788	0.313	0.982
**PC-IV - PC-I**	0.776	0.982	0.782	0.751
**PC-II - MC-II**	0.619	0.112	0.219	0.082
**MC-III - MC-II**	0.884	1.000	0.999	0.967
**PC-III - MC-II**	0.648	0.964	0.999	0.262
**MC-IV - MC-II**	0.173	1.000	1.000	0.808
**PC-IV - MC-II**	0.397	0.979	1.000	0.408
**MC-III - PC-II**	1.000	0.098	0.076	0.466
**PC-III - PC-II**	1.000	0.571	0.519	0.998
**MC-IV - PC-II**	0.985	0.203	0.094	0.749
**PC-IV - PC-II**	1.000	0.509	0.385	0.981
**PC-III - MC-III**	1.000	0.950	0.941	0.833
**MC-IV - MC-III**	0.858	1.000	1.000	1.000
**PC-IV - MC-III**	0.987	0.970	0.982	0.941
**MC-IV - PC-III**	0.980	0.996	0.964	0.975
**PC-IV - PC-III**	1.000	1.000	1.000	1.000
**PC-IV - MC-IV**	0.999	0.998	0.991	0.997

Table S2. Two-way PERMANOVA of cultural stages and modes for bacterioplankton based on the Bray-Curtis distance.

	F	R^2	p-value
**Stage**	9.1709	0.4234	0.001
**Mode**	4.3882	0.0675	0.001
**Stage:Mode**	3.0237	0.1396	0.001

Table S3. PERMANOVA of cultural modes for bacterioplankton in each cultural stage based on the Bray-Curtis distance.

	F	R^2	p-value
**Stage-I**	2.3179	0.2787	0.090
**Stage-II**	5.9636	0.4985	0.027
**Stage-III**	3.0148	0.3344	0.035
**Stage-IV**	2.2019	0.2685	0.054

Table S4. The p-values of Tukey's HSD tests for environmental factors in cultured ponds among different stages with and without the apex predator species.

Group1 – Group2	TP	TN	Ammonia	Nitrite	Nitrate	pH	DO	Temperature
**PC-I - MC-I**	1.000	0.740	0.257	1.000	0.109	0.000	0.000	1.000
**MC-II - MC-I**	1.000	0.996	0.000	0.000	0.000	0.274	0.998	0.000
**PC-II - MC-I**	1.000	0.837	0.000	0.990	0.000	0.210	0.096	0.000
**MC-III - MC-I**	1.000	0.025	0.000	0.055	0.022	0.447	0.124	0.000
**PC-III - MC-I**	1.000	0.019	0.000	0.049	0.998	0.315	0.333	0.000
**MC-IV - MC-I**	0.960	0.291	0.016	0.626	1.000	0.388	0.995	0.000
**PC-IV - MC-I**	0.999	0.747	0.348	0.524	0.958	0.398	0.956	0.000
**MC-II - PC-I**	1.000	0.333	0.000	0.000	0.000	0.000	0.000	0.000
**PC-II - PC-I**	1.000	0.089	0.000	1.000	0.000	0.000	0.010	0.000
**MC-III - PC-I**	1.000	0.001	0.000	0.120	0.000	0.000	0.007	0.000
**PC-III - PC-I**	1.000	0.000	0.000	0.108	0.027	0.000	0.002	0.000
**MC-IV - PC-I**	0.991	0.011	0.866	0.396	0.201	0.000	0.000	0.000
**PC-IV - PC-I**	1.000	0.063	1.000	0.311	0.010	0.000	0.000	0.000
**PC-II - MC-II**	1.000	0.995	0.954	0.000	0.014	1.000	0.294	0.996
**MC-III - MC-II**	1.000	0.111	0.000	0.000	0.753	1.000	0.359	0.996
**PC-III - MC-II**	1.000	0.086	0.207	0.000	0.002	1.000	0.699	1.000
**MC-IV - MC-II**	0.987	0.689	0.004	0.000	0.000	1.000	0.867	0.023
**PC-IV - MC-II**	1.000	0.983	0.000	0.000	0.007	1.000	0.689	0.036
**MC-III - PC-II**	0.997	0.391	0.001	0.264	0.000	1.000	1.000	1.000
**PC-III - PC-II**	0.999	0.325	0.795	0.241	0.000	1.000	0.996	0.996
**MC-IV - PC-II**	0.998	0.975	0.000	0.196	0.000	1.000	0.019	0.106
**PC-IV - PC-II**	1.000	1.000	0.000	0.146	0.000	1.000	0.009	0.156
**PC-III - MC-III**	1.000	1.000	0.035	1.000	0.091	1.000	0.999	0.996
**MC-IV - MC-III**	0.893	0.916	0.000	0.001	0.010	1.000	0.026	0.106
**PC-IV - MC-III**	0.991	0.492	0.000	0.001	0.201	1.000	0.012	0.157
**MC-IV - PC-III**	0.929	0.869	0.000	0.001	0.974	1.000	0.089	0.023
**PC-IV - PC-III**	0.996	0.417	0.000	0.000	1.000	1.000	0.043	0.036
**PC-IV - MC-IV**	1.000	0.992	0.771	1.000	0.853	1.000	1.000	1.000

Table S5. Significance tests of environmental factors in db-RDA.

	R^2	p-value
**TP**	0.0124	0.833
**TN**	0.5343	0.001
**Ammonia**	0.4597	0.001
**Nitrite**	0.0801	0.288
**Nitrate**	0.7591	0.001
**pH**	0.5278	0.001
**DO**	0.0982	0.204
**Temperature**	0.8389	0.001

Table S6. Topological parameters of molecular ecology networks constructed by bacterioplankton in cultured ponds with and without the apex predator species.

Topological parameters	MC	PC
**Node number**	119	98
**Edge number**	295	256
**Average degree**	4.958	5.224
**Diameter**	13	13
**Density**	0.042	0.054
**Modularity**	0.716	0.678

Table S7. Results of linear regression for variations in environmental factors with the phylogenetic distances between different samples.

Environmental factor	Cultural mode	slope	R2	p-value
**Temperature**	MC	0.001	0.313	1.93E-11
	PC	0.001	0.090	0.001
**pH**	MC	0.013	0.281	2.81E-10
	PC	0.002	0.016	0.089
**DO**	MC	0.000	-0.008	0.860
	PC	0.002	0.018	0.076
**Ammonia**	MC	0.015	0.167	2.12E-06
	PC	0.011	0.030	0.032
**Nitrate**	MC	0.001	-0.008	0.885
	PC	0.034	0.292	1.10E-10
**Nitrite**	MC	0.014	-0.006	0.620
	PC	-0.001	-0.008	0.987
**TN**	MC	0.001	0.061	0.004
	PC	0.001	0.006	0.197
**TP**	MC	0.067	0.016	0.088
	PC	-0.017	-0.006	0.600
